# Associations Between Physical Activity and Hypertension in Chinese Children: A Cross-Sectional Study From Chongqing

**DOI:** 10.3389/fmed.2021.771902

**Published:** 2021-12-15

**Authors:** Qianqian Wang, Ping Qu, Jingyu Chen, Xian Tang, Guang Hao, Xiaohua Liang

**Affiliations:** ^1^Department of Epidemiology and Biostatistics, Institute of Traumatology and Orthopaedics, Beijing Jishuitan Hospital, Beijing, China; ^2^Department of Clinical Epidemiology and Biostatistics, Children's Hospital of Chongqing Medical University, National Clinical Research Center for Child Health and Disorders, Ministry of Education Key Laboratory of Child Development and Disorders, Chongqing Key Laboratory of Child Health and Nutrition, Chongqing, China; ^3^Department of Epidemiology, School of Medicine, Jinan University, Guangzhou, China

**Keywords:** physical activity, hypertension, child, risk factors, cross-sectional study

## Abstract

**Background:** Childhood blood pressure is a marker for cardiovascular disease risk later in life. Few studies examined the association between physical activity (PA) and hypertension in Chinese children, and this study aimed to explore this relationship.

**Methods:** A cross-sectional study among pupils was conducted in Chongqing in 2014. In total, 15,203 children aged 6–12 years in Chongqing were included in this study. The duration of self-reported PA on school days and the weekend in pupils were collected with a standardized questionnaire.

**Results:** The results showed that, on school days, only 22.3% of boys and 17.8% of girls engaged in more than 60 min of PA per day; while on the weekend, only 38.5% of boys and 32.0% of girls engaged in at least 60 min of PA per day. There was no strong evidence for an association between PA and systolic or diastolic hypertension in boys. However, in girls, a weak negative association between PA on weekdays and hypertension was observed, and there was a significant interactive effect of PA and obesity on hypertension risk (*P*_for interaction_ = 0.042). In obese children, PA was positively related to the risk of hypertension.

**Conclusion:** The overall level of PA among pupils in Chongqing was insufficient, and a weak negative relationship between PA and hypertension was found in girls.

## Introduction

Hypertension is one of the most common causes of cardiovascular disease and mortality worldwide (1). The prevalence of hypertension is increasing globally because of the aging of the population and growing unhealthy lifestyles such as a lack of physical activity (PA) (2). Previous evidence suggests that elevated blood pressure (BP) in children is associated with a high risk of cardiovascular disease in later life (3–7). Based on the use of three or more BP measurements in the ≥95th percentile, the prevalence of pediatric hypertension is estimated to range from 1 to 3% (8). As a result of the childhood obesity epidemic, increasing numbers of children are being diagnosed with hypertension (9, 10). Moreover, because the cutoffs for pediatric hypertension are based on standard deviations from normative BP data and expert opinion, hypertension and prehypertension may be underdiagnosed in the pediatric population (11).

Although childhood hypertension shares some risk factors similar to hypertension in adults, hypertension in children and adolescents aged ≤ 16 years has a variety of risk factors that differ from those of adults because of specific events that occur during the growth and development of children (e.g., maternal factors and birth weight) (12, 13). It is crucial to examine the modifiable factors that may be associated with rising BP in children. One such potential factor is PA. Physical inactivity is positively associated with higher BP and cardiovascular disease in adulthood (14), and regular PA has the potential to lower BP and weight among adults (15–17). Unlike in adults, the association between PA and BP in children remains controversial. Several longitudinal studies and one review revealed an inverse association between PA and BP (18–21). However, several prospective studies have shown no significant association between PA and BP in children (22, 23). To date, the majority of such studies have been conducted in Western countries, and little information is available from low- and middle-income countries. The purposes of the present study were to examine the levels of PA in children aged 6–12 years and to explore the association between PA and hypertension in China.

## Materials and Methods

### Study Description

This cross-sectional study was conducted in Chongqing in 2014. A detailed description of the study has been published elsewhere (24). Briefly, two-stage stratified cluster sampling was used to include three counties in Chongqing (first stratification) and two randomly selected avenues per country (second stratification). All children aged 6–12 years living in the selected avenues were included in this study. All eligible participants finished the questionnaires, which included questions on diet, socioeconomic status, and family health history. Finally, 18,054 children in the selected avenues provided written informed consent, and 17,078 subjects participated in the physical examination and survey questionnaire. After excluding 71 participants with missing information regarding age, sex, height, and BP; 17,007 participants remained. Additionally, 1,804 participants had missing information regarding PA; therefore, 15,203 participants were ultimately included. A flow chart of the participants' inclusion and exclusion process is shown in Supplementary Figure 1. The analyzed sample (15,203 participants) is representative of the original population (17,007 subjects), and the baseline characteristics of the two samples are listed in Supplementary Table 1.

### Physical Activity Measurements

PA was assessed using self-reported questionnaires and was recorded separately for weekdays and the weekend. For each PA reported, the most frequent activity category, including heavy PA (e.g., playing basketball and badminton, swimming, hiking, and fast running), medium PA (e.g., slow running, dancing, and cycling), light PA (e.g., walking, strength exercises, doing housework, and playing ping-pong), and sedentary lifestyle (e.g., watching TV, reading, playing computer games, and doing school work), and the corresponding the number of minutes per day were obtained (24). Light PA was scored with a coefficient of 1, and the coefficients of the average metabolic intensities of heavy PA, medium PA, and sedentary lifestyle were 2.0, 1.5, and 0.3, respectively, as estimated based on data from the youth compendium of PA (25). The final duration of PA was equal to the sum of the time spent at a given level of PA multiplied by the corresponding coefficient. Total PA per week was calculated by adding the duration of PA on weekdays to the duration of PA on weekends. Information regarding PA was collected with the cooperation of the parents, teachers, and children to ensure the reliability of the PA information.

### BP Measurements

BP and heart rate were measured on three separate occasions using an arm-type electronic sphygmomanometer (HEM-7051; OMRON, Kyoto, Japan), and the details of how measurements were performed are described in a previous publication (24, 26, 27). Briefly, BP measurements were taken at 11, 13, and 15 min with the subject sitting down during a 15-min relaxation period in the morning (09:00–12:00), using an appropriately sized BP cuff on the subject's right arm. The average value of the three BP measurements was used to represent the resting systolic BP (SBP) and diastolic BP (DBP). If the first BP screening met the criteria for hypertension, the second and third measurements were conducted in the following 2–4 weeks. Secondary hypertension was excluded by a pediatrician through an interview regarding the subject's medical history and by performing physical examinations on hypertensive participants in stage two. The hypertension diagnostic criteria described by Mi Jie (28) were considered suitable for the growth characteristics of children and teenagers in China and were used in this study. Hypertension was defined as a mean measured SBP and/or DBP in the ≥95th percentile, based on age, sex, and height percentiles. Participants were diagnosed with hypertension if all three BP measurements on three occasions on different days met the criteria for hypertension.

### Covariates

The confounders were variables that were reported to be significantly associated with the risk of hypertension in a previous study (18). The covariates included in the multivariable analyses were demographic information consisting of age and living environment (urban, suburban, or rural); prenatal variables including birth weight, breastfeeding, and pregnancy-induced hypertension; anthropometric variables such as heart rate and body mass index (BMI); and dietary information (e.g., cereals and potatoes, pickles). Anthropometric measurements were conducted by well-trained pediatric nurses. Height and weight were measured using a mobile medical ultrasonic machine (model WS-H300D), and BMI was calculated as weight (kg) divided by height squared (m^2^) as a measure of general adiposity. Being overweight was defined as a BMI from the 85th to <95th percentile and obesity was defined as a BMI in the ≥95th percentile according to the sex-specific BMI-for-age growth charts established by the Centers for Disease Control and Prevention (29). A previously reported quantitative food frequency questionnaire was used to collect dietary information (26). The questionnaire was completed by the parents or guardians of the children after standard training by the research group.

### Data Analyses

Continuous variables are presented as the means ± standard deviation, and the means of two groups were compared using Student's *t*-test. Categorical variables are reported as percentages (n [%]), and the χ^2^ test was used to test differences between groups. All PA variables were divided into tertiles for all analyses. Unadjusted and adjusted analyses for the associations between PA (categorized into tertiles) and diagnosed hypertension (dichotomous) were performed to establish odds ratios (ORs) with corresponding confidence intervals (CIs). The reference category was set as the lowest (least active) tertile. The associations were tested using three sets of statistical models: Model 1 was the unadjusted analysis of the association between PA and hypertension; Model 2 was the same as Model 1 plus adjustments for age and BMI; and Model 3 included additional adjustments for the region, birth weight, breastfeeding, pregnancy-induced hypertension, heart rate, and intake of cereals, potatoes, and pickles. Data were stratified by age, region, birthweight, and BMI for subgroup analyses, and potential interactions were also tested. The complex cross-sectional survey sampling design was used because individuals were clustered into schools. Therefore, the GLIMMIX procedure was used to fit the two-level (individuals and schools) logistic regression mixed models in which schools were treated as clusters. An unconditional logistic regression model was used in the subgroup analyses because of the sparseness of data in some subgroups.

The data analysis was conducted using SAS 9.4 software (SAS Institute Inc. Cary, NC, USA). A significant difference was defined by an α level of 0.05 in two-sided tests.

## Results

### Characteristics of the Study Population

In total, 15,203 children (7,899 [51.96%] boys) were included in the current analysis. The characteristics of the participants are summarized in Table 1. The mean age of the participants was 9.30 ± 1.74 years for boys and 9.19 ± 1.74 years for girls. The mean SBP and DBP were 103.9 ± 9.9 and 63.0 ± 7.7 mmHg for boys and 102.2 ± 9.9 and 63.4 ± 7.5 mmHg for girls, respectively. The prevalence of hypertension was 13.2 and 14.0%, respectively (Table 1).

**Table 1:** Characteristics of included subjects.

**Variables**	**Boys** **(***N*** = 7899)**	**Girls** **(***N*** = 7304)**
**Age (years)**	9.3 ± 1.7	9.2 ± 1.7
**Region**		
Urban	28.9% (2280/7899)	30.5% (2224/7304)
Rural	48.4% (3821/7899)	48.1% (3510/7304)
Suburb	22.8% (1798/7899)	21.5% (1570/7304)
**Birthweight (kg)**	6.6 ± 1.1	6.4 ± 1.1
**Breastfeeding (month)**		
0~3	24.4% (1890/7737)	25.6% (1839/7195)
4~10	48.4% (3743/7737)	50.5% (3634/7195)
>10	27.2% (2104/7737)	23.9% (1722/7195)
**Gestational hypertension**	1.6% (118/7622)	1.5% (104/7089)
**Cereals and potatoes (g/day)**	171(100, 250)	150(100, 250)
**Intake of pickles (g/day)**	7.1(0.3, 18.3)	7.1(0.7, 16.7)
**SBP (mmHg)**	103.9 ± 9.9	102.2 ± 9.9
**DBP (mmHg)**	63.0 ± 7.7	63.4 ± 7.5
**Prevalence of systolic hypertension,%(n)**	8.8% (696/7899)	8.3% (609/7304)
**Prevalence of diastolic hypertension,%(n)**	7.8% (615/7899)	9.5% (692/7304)
**Prevalence of hypertension,%(n)**	13.2% (1039/7899)	14.0% (1019/7304)

*Data are presented as mean ± standard deviation, % (n), or median (quantiles). SBP, systolic blood pressure; DBP, diastolic blood pressure*.

### PA in the Study Population

The median amount of PA on weekdays and on the weekend were 9.0 (3.0, 45.0) and 30.0 (6.0, 60.0) min/day in boys and 7.5 (3.0, 40.0) and 30.0 (6.0, 60.0) min/day in girls, respectively. On school days, only 22.3% of boys and 17.8% of girls engaged in more than 60 min of PA per day. Even on the weekend, only 38.5% of boys and 32.0% of girls engaged in at least 60 min of PA per day. Generally, boys engaged in more PA than girls (Table 2).

**Table 2:** Physical activity levels in participants.

**PA variable**	**Boys**	**Girls**
**Physical activity (median, quantiles)**		
Weekday, min/day	9.0(3.0, 45.0)	7.5(3.0, 40.0)
Weekend, min/day	30.0(6.0, 60.0)	30.0(6.0, 60.0)
**Physical activity (low tertile, median tertile, upper tertile)**		
*Weekday, min/day*		
First tertile (Q1)	0~4.0	0~4.0
Second tertile (Q2)	4.1~30.0	4.1~30.0
Third tertile (Q3)	>30.0	>30.0
*Weekend, min/day*		
First tertile (Q1)	0~20.0	0~20.0
Second tertile (Q2)	20.1~60.0	20.1~45.0
Third tertile (Q3)	>60.0	>45.0
*Total PA, min/week*		
First tertile (Q1)	0~84.0	0~75.0
Second tertile (Q2)	84.1~270.0	75.1~231.0
Third tertile (Q3)	>270.0	>231.0
**More than 60 min per day,%**		
Weekday	22.3% (1760/7899)	17.8% (1303/7304)
Weekend	38.5% (3030/7877)	32.0% (2334/7293)
**More than 120 min per day, %**		
Weekday	6.5% (512/7899)	5.0% (367/7304)
Weekend	11.6% (916/7899)	8.6% (625/7304)

*PA, physical activity*.

### Association Between Hypertension and PA

No strong evidence was found for associations between PA and systolic or diastolic hypertension in boys in any of the models (Table 3). Among girls, there was weak evidence for an inverse association between PA on weekdays (Model 3) and hypertension when comparing the third tertile (Q3) of PA with the first tertile (Q1) (Table 4). Furthermore, when comparing the Q2 level of total PA with the Q1 level, a protective effect of total PA against diastolic hypertension was found in Model 3 (Supplementary Table 2).

**Table 3:** Logistic mixed regression of relationship between physical activity and hypertension in boys.

**PA variable**	**Systolic hypertension**			**Diastolic hypertension**			**Hypertension**		
	**Model 1**	**Model 2**	**Model 3**	**Model 1**	**Model 2**	**Model 3**	**Model 1**	**Model 2**	**Model 3**
**Physical activity on weekdays (Ref. Q1)**									
Q2 VS. Q1	1.02 (0.83, 1.26)	1.01 (0.82, 1.25)	0.97 (0.24, 3.84)	1.02 (0.82, 1.26)	1.05 (0.84, 1.30)	0.99 (0.80, 1.23)	1.02 (0.86, 1.21)	1.02 (0.86, 1.22)	0.98 (0.82, 1.17)
Q3 VS. Q1	0.98 (0.79, 1.22)	0.93 (0.74, 1.18)	0.96 (0.21, 4.36)	0.94 (0.75, 1.19)	1.02 (0.80, 1.29)	1.10 (0.87, 1.39)	0.95 (0.79, 1.15)	0.96 (0.79, 1.16)	1.01 (0.83, 1.22)
**Physical activity on the weekend (Ref. Q1)**									
Q2 VS. Q1	1.05 (0.86, 1.27)	1.03 (0.84, 1.25)	1.05 (0.28, 3.98)	1.07 (0.87, 1.31)	1.06 (0.86, 1.30)	1.04 (0.84, 1.28)	1.05 (0.89, 1.24)	1.03 (0.87, 1.22)	1.07 (0.90, 1.26)
Q3 VS. Q1	1.08 (0.84, 1.39)	1.01 (0.78, 1.31)	1.15 (0.22, 6.12)	0.86 (0.65, 1.14)	0.90 (0.68, 1.19)	0.98 (0.75, 1.29)	1.08 (0.87, 1.33)	1.07 (0.86, 1.32)	1.22 (0.98, 1.51)

*Data are presented as odds ratio (95% confidence interval). PA, physical activity, Q1, the first tertile level of PA, Q2, the second tertile level of PA, Q3, the third tertile level of PA. Model 1: unadjusted; Model 2: adjusted for age and BMI; Model 3: adjusted for age, region, BMI, heart rate, breastfeeding, birth weight, gestational hypertension, intake of cereals and potatoes, and intake of pickles*.

**Table 4:** Logistic mixed regression of relationship between physical activity and hypertension in girls.

**PA variable**	**Systolic hypertension**			**Diastolic hypertension**			**Hypertension**		
	**Model 1**	**Model 2**	**Model 3**	**Model 1**	**Model 2**	**Model 3**	**Model 1**	**Model 2**	**Model 3**
**Physical activity on weekdays (Ref. Q1)**									
Q2 VS. Q1	0.94 (0.76, 1.16)	0.90 (0.72, 1.12)	0.87 (0.21, 3.53)	0.90 (0.74, 1.10)	0.92 (0.75, 1.12)	0.86 (0.71, 1.06)	0.92 (0.77, 1.08)	0.91 (0.76, 1.08)	0.87 (0.73, 1.03)
Q3 VS. Q1	0.91 (0.72, 1.15)	0.89 (0.70, 1.13)	0.85 (0.18, 4.05)	0.82 (0.65, 1.02)	0.85 (0.67, 1.06)	0.82 (0.65, 1.03)	0.85 (0.70, 1.03)	0.85 (0.70, 1.03)	0.82 (0.68, 1.00)^*^
**Physical activity on the weekend (Ref. Q1)**									
Q2 VS. Q1	1.05 (0.84, 1.32)	1.08 (0.85, 1.36)	1.13 (0.25, 5.14)	1.10 (0.89, 1.35)	1.09 (0.88, 1.34)	1.12 (0.91, 1.38)	1.06 (0.89, 1.27)	1.07 (0.89, 1.28)	1.10 (0.92, 1.32)
Q3 VS. Q1	1.14 (0.92, 1.42)	1.11 (0.88, 1.39)	1.18 (0.27, 5.07)	0.84 (0.67, 1.04)	0.88 (0.71, 1.09)	0.90 (0.73, 1.12)	0.94 (0.78, 1.12)	0.94 (0.79, 1.13)	0.98 (0.82, 1.17)

*Data are presented as odds ratio (95% confidence interval)*.

* *P < 0.05*

*PA, physical activity, Q1, the first tertile level of PA, Q2, the second tertile level of PA, Q3, the third tertile level of PA. Model 1: unadjusted. Model 2: adjusted by age and BMI. Model 3: adjusted by age, region, BMI, heart rate, breastfeeding, birth weight, gestational hypertension, intake of cereals and potatoes, and intake of pickles*.

**Figure 1:**
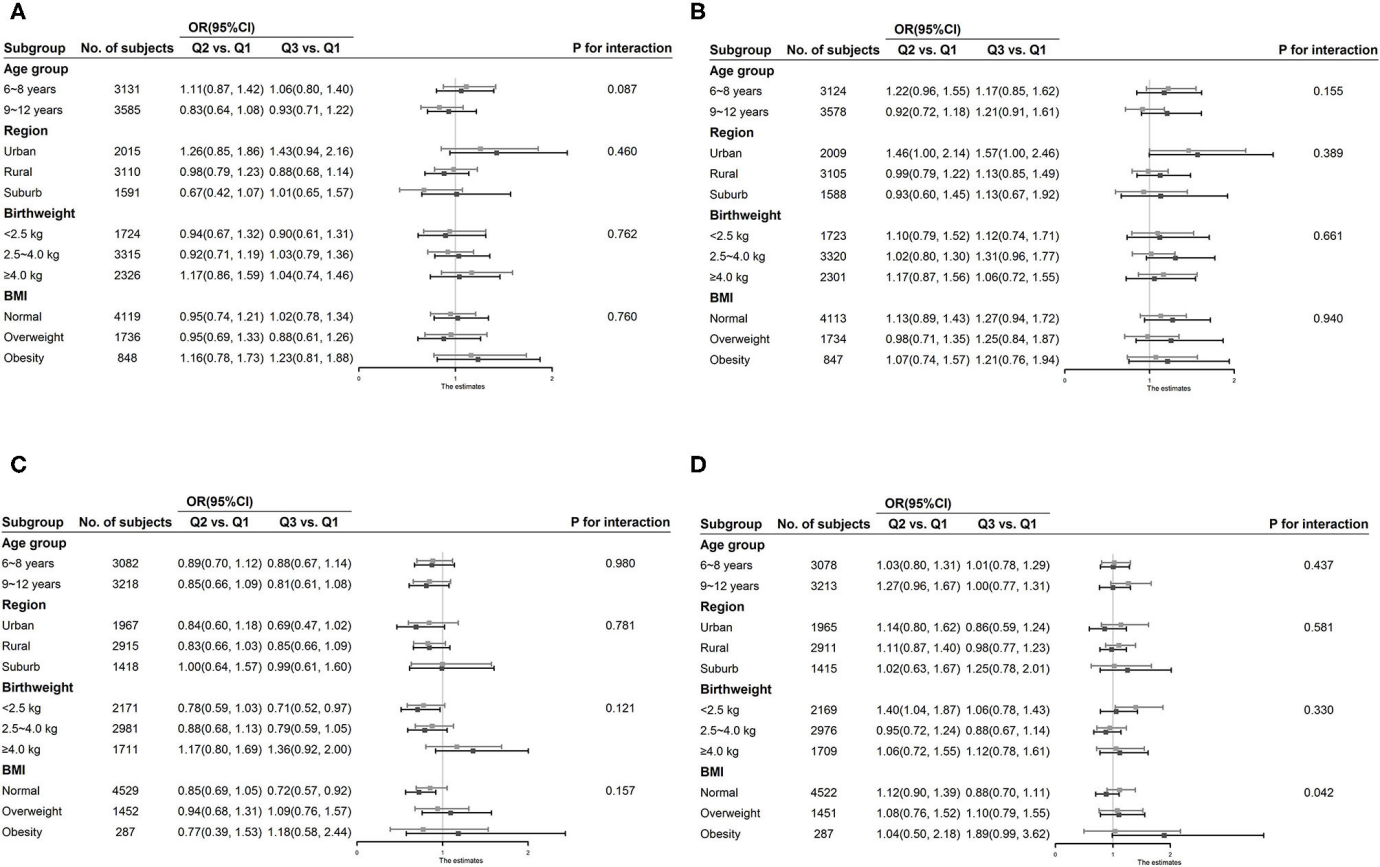
Subgroup analysis of association between physical activity and hypertension stratified by age, region, birthweight, and body mass index. **(A)** Association of physical activity on weekdays with hypertension in boys. **(B)** Association of physical activity on the weekend with hypertension in boys. **(C)** Association of physical activity on weekdays with hypertension in girls. **(D)** Association of physical activity on the weekend with hypertension in girls.

No significant relationship was found between PA on weekdays or the weekend and hypertension for boys in the subgroup analysis stratified by age, region, birthweight, and BMI. No interactions were identified between PA and age, region, birth weight, or BMI for hypertension in boys. In girls, however, we found a significant interactive effect of PA and obesity on hypertension risk (*P*_for interaction_ = 0.042). In obese participants, PA was positively related to the risk of hypertension. No significant results were discovered between the other subgroups (Figure 1).

## Discussion

Our results showed that the overall level of PA among pupils in Chongqing was insufficient. The amount of PA in almost 80% of the participants was <60 min/ day. The time spent performing moderate-to-vigorous PA maybe even less because the pattern of activity was standardized to light PA in the present study. This is consistent with evidence from large-scale cross-sectional and retrospective population studies in China (30). These findings emphasize the need for the implementation of public health interventions and policies that will substantially improve the PA levels of most children and adolescents.

Generally, no significant association was found between PA and hypertension in boys in the present study. This differs from the results of a study by Kong et al.'s (31), who found that the self-reported PA levels were independently associated with cardiovascular risk. However, their study had a relatively small sample size of 2,119 Hong Kong Chinese youths aged 6–20 years. In addition, cardiovascular risk was quantified using a summary risk score comprising waist circumference, BP, fasting plasma glucose, and lipids. Moreover, only a few risk factors (sex, age, and puberty) were adjusted for in the regression model.

In the present study, the benefits of PA were probably not identified because of the overall low level and intensity of PA in this population. Although the international guidelines on PA for children and adolescents (age range of 5–17 years) generally recommend an active lifestyle and encourage daily PA participation, including all intensities of PA (32), physical activities of vigorous-intensity are commonly recommended by most guidelines because they are more favorably related to a variety of health outcomes in children and adolescents (21, 33). A study by Mark and Janssen (34) (including 1,170 children aged 8–17 years from 2003 to 2004 U.S. National Health and Nutrition Examination Survey) showed that the risk of hypertension was reduced by ~50% at 30 min/day of moderate-to-vigorous PA vs. 90 min/day of total PA. Thus, in the development of strategies aimed at preventing and treating hypertension in children and adolescents, both the duration and intensity of PA are important factors that should be considered.

An alternative perspective is that PA may not be a key factor influencing BP in childhood, and that other determinants, such as obesity and genetic influences, maybe more important risk factors for higher blood pressure in the first decade of life (22). The results of a cross-sectional Danish study that included 589 children aged 8–10 years, showed no significant evidence of an association between accelerometer-assessed moderate-to-vigorous-intensity PA and SBP or DBP (35). In the B-PROACT1V, a longitudinal study of 1,223 children from Bristol, UK, there was no evidence that PA or sedentary time at 6 or 9 years was cross-sectionally or prospectively associated with BP at 9 years. Updated findings from the B-PROACT1V study showed similar trends for PA and BP (23). The present study added to this evidence, presenting a lack of strong evidence for an association between PA and hypertension, especially in boys.

Among girls, a weak negative association between PA on weekdays and hypertension was found in this study. As indicated by Kong et al. (31), the sex-related difference might be the result of sex-related differences in lifestyle, energy expenditure, body fat composition, and hormonal levels. For girls, there was a significant interactive effect of PA and obesity on hypertension risk. A positive relationship between PA and hypertension was observed in obese girls. However, this relationship might be due to reverse causality. The available evidence has generally demonstrated a strong positive relationship between BMI and BP in children and adolescents (36, 37), and the prevalence of hypertension in overweight/obese children was significantly higher than that in children with a healthy weight (38). In practice, obese children might be urged by their parents to engage in more PA, which may create the illusion of a significant association between PA and increased blood pressure in obese subjects.

A limitation of this study is its cross-sectional design, which prevent us from drawing conclusions regarding causality. Moreover, self-reported PA is subject to recall bias and misclassification, which also prevented us from evaluating the effect of PA intensity on BP and may dilute the true effect of PA on BP. A prospective birth cohort study of 427 11 to 14-year-old children in Pelotas, Brazil showed that accelerometry-based PA, but not self-reported PA, was longitudinally inversely associated with DBP (39). Moreover, other potentially BP-associated factors, such as genetics, were not considered in this analysis.

## Conclusions

Our results showed that the overall level of PA among pupils in Chongqing was insufficient, and a weak negative relationship between PA and hypertension was found in girls. Further longitudinal studies with an objective PA measure are needed to accurately clarify the causal relationship between PA and hypertension in Chinese children.

## Data Availability Statement

The data analyzed in this study is subject to the following licenses/restrictions: The datasets generated and/or analyzed during the current study are not publicly available due to privacy and confidentiality agreements as well as other restrictions but are available from the corresponding author on reasonable request. Requests to access these datasets should be directed to XL (xiaohualiang@hospital.cqmu.edu.cn).

## Ethics Statement

The studies involving human participants were reviewed and approved by Institutional Review Board at the Children's Hospital of Chongqing Medical University. Written informed consent to participate in this study was provided by the participants' legal guardian, and next of kin.

## Author Contributions

XL conceived and designed the experiments and revised the manuscript. QW performed data analysis and wrote the paper. PQ, JC, and XT performed the experiments. XL and JC participated in the physical measurements. GH reviewed and edited manuscript. All authors critically reviewed and approved the final paper.

## Funding

This work was supported by the Intelligent Medicine Project (ZHYX202109), Basic Research Project of Key Laboratory of Ministry of Education of China in 2021 (GBRP-202106), Joint Medical Research Project of Chongqing Municipal Health Commission and Chongqing Science and Technology Bureau (2020MSXM062), National Key Research and Development Project (2017YFC0211705), Education Commission of Chongqing Municipality (KJQN201900443), and National Natural Science Foundation of China (81502826).

## Conflict of Interest

The authors declare that the research was conducted in the absence of any commercial or financial relationships that could be construed as a potential conflict of interest.

## Publisher's Note

All claims expressed in this article are solely those of the authors and do not necessarily represent those of their affiliated organizations, or those of the publisher, the editors and the reviewers. Any product that may be evaluated in this article, or claim that may be made by its manufacturer, is not guaranteed or endorsed by the publisher.

## Supplementary Material

Supplementary Figure 1 Flow chart of participants, inclusion and exclusion criteria.

Supplementary Table 1 The baseline characteristics of the two populations (17,007 vs. 15,203).

Supplementary Table 2 Logistic mixed regression (odds ratio and 95% confidence interval) of the relationship between total physical activity and hypertension.
