# Supplementary figures and images for: Associations Between Physical Activity and Hypertension in Chinese Children: A Cross-Sectional Study From Chongqing
Source: Front Med (Lausanne). 2021 Dec 15;8:771902. doi: 10.3389/fmed.2021.771902 (PMC8714888; doi:10.3389/fmed.2021.771902)

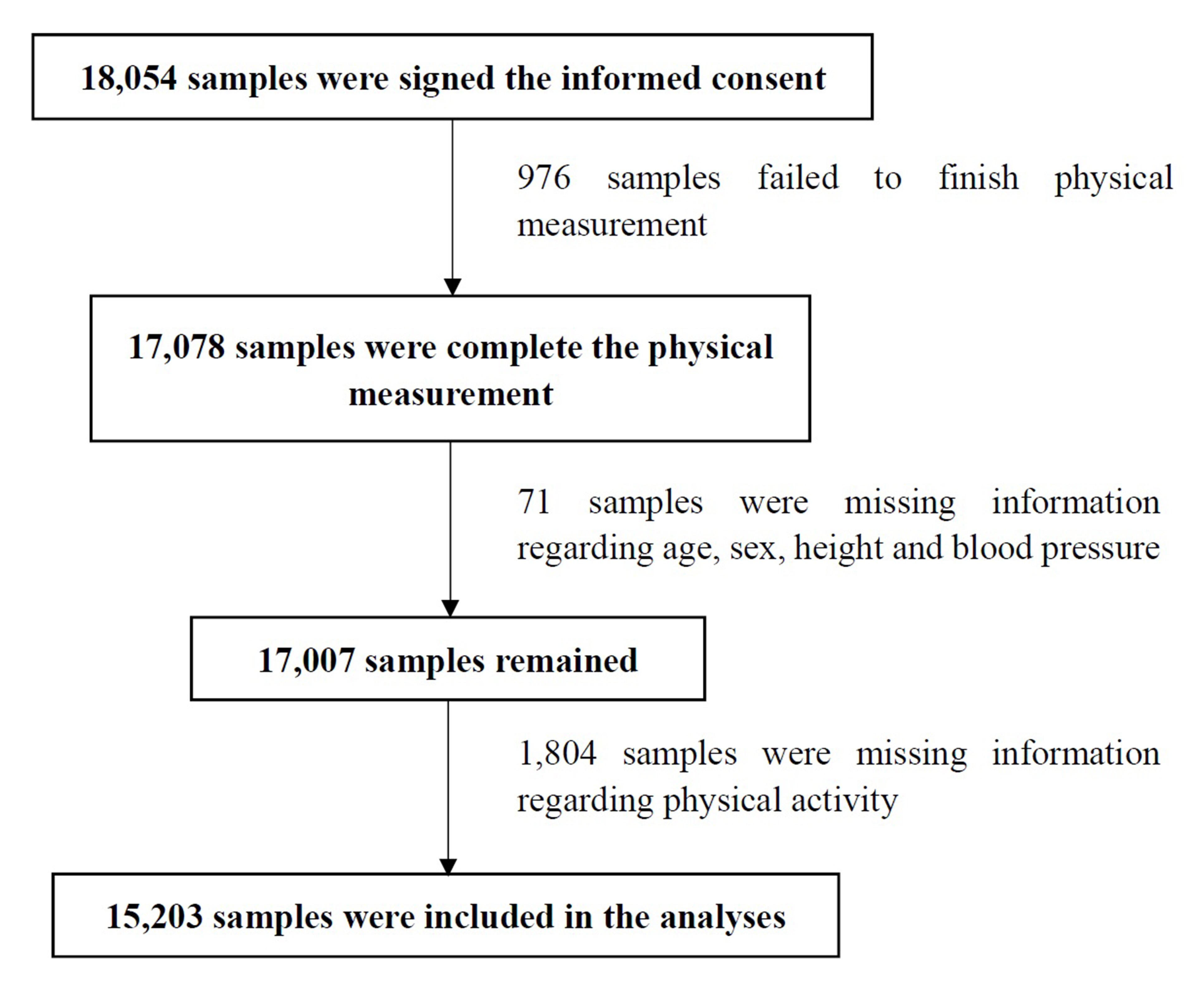

Supplement: Supplementary Figure 1 — Flow chart of participants, inclusion and exclusion criteria. [file Image_1.JPEG]
