# Supplementary material for: Associations Between Physical Activity and Hypertension in Chinese Children: A Cross-Sectional Study From Chongqing
Source: Front Med (Lausanne). 2021 Dec 15;8:771902. doi: 10.3389/fmed.2021.771902 (PMC8714888; doi:10.3389/fmed.2021.771902)
Supplement: Supplementary Table 1 — The baseline characteristics of the two populations (17,007 vs. 15,203). [file Table_1.DOCX]

**Supplementary Table 1. The baseline characteristics of the two populations (17,007 vs. 15,203).**

|  | **17,007**  **samples** | **15,203**  **samples** | **T value**  **/χ^2^** | ***P* value** |
| --- | --- | --- | --- | --- |
| **Age (years)** | 9.20±1.74 | 9.25±1.74 | -2.65 | 0.008 |
| **Sex (male/female)** | 8881/8126 | 7899/7304 | 0.22 | 0.637 |
| **Height (cm)** | 134.4±11.4 | 134.7±11.4 | -1.90 | 0.058 |
| **Weight (kg)** | 31.99±9.50 | 32.13±9.54 | -1.32 | 0.187 |

**Supplementary Table 2. Logistic mixed regression (odds ratio and 95% confidence interval) of the relationship between total physical activity and hypertension**

| Total PA, min/week | Systolic Hypertension | | |  | Diastolic Hypertension | | |  | Hypertension | | |
| --- | --- | --- | --- | --- | --- | --- | --- | --- | --- | --- | --- |
|  | Model 1 | Model 2 | Model 3 |  | Model 1 | Model 2 | Model 3 |  | Model 1 | Model 2 | Model 3 |
| **Boys** | | |  |  |  |  |  |  |  |  |  |
| Q2 VS. Q1 | 0.97(0.79, 1.20) | 0.94(0.76, 1.17) | 1.00(0.25, 4.02) | | 0.95(0.76, 1.18) | 0.96(0.77, 1.20) | 0.96(0.77, 1.20) |  | 0.99(0.83, 1.18) | 0.98(0.82, 1.17) | 1.04(0.87, 1.24) |
| Q3 VS. Q1 | 0.97(0.78, 1.21) | 0.91(0.73, 1.15) | 1.00(0.22, 4.53) | | 0.90(0.71, 1.13) | 0.96(0.76, 1.22) | 1.03(0.81, 1.31) |  | 0.96(0.79, 1.15) | 0.95(0.79, 1.16) | 1.06(0.87, 1.28) |
| **Girls** | | |  |  |  |  |  |  |  |  |  |
| Q2 VS. Q1 | 1.02(0.81, 1.27) | 1.01(0.80, 1.27) | 1.03(0.24, 4.44) | | 0.82(0.67, 1.02) | 0.83(0.67, 1.03) | 0.79(0.64, 0.98)^*^ |  | 0.87(0.73, 1.04) | 0.87(0.72, 1.04) | 0.84(0.70, 1.01) |
| Q3 VS. Q1 | 0.98(0.78, 1.23) | 0.94(0.74, 1.18) | 0.94(0.21, 4.25) | | 0.83(0.67, 1.02) | 0.86(0.69, 1.07) | 0.85(0.68, 1.05) |  | 0.88(0.73, 1.05) | 0.87(0.73, 1.05) | 0.86(0.72, 1.03) |

***, *P*<0.05**

**PA: physical activity, Q1: the first tertile level of PA, Q2: the second tertile level of PA, Q3: the third tertile level of PA.** Model 1: unadjusted. Model 2: adjusted by age and BMI. Model 3: adjusted by age, region, BMI, heart rate, breast feeding, birth weight, gestational hypertension, intake of cereals and potatoes, intake of pickles.
